# Experimental Analysis of Fiber Reinforcement Rings’ Effect on Tensile and Flexural Properties of Onyx™–Kevlar^®^ Composites Manufactured by Continuous Fiber Reinforcement

**DOI:** 10.3390/polym15051252

**Published:** 2023-03-01

**Authors:** Benjamín Alberto Moreno-Núñez, César Gustavo Abarca-Vidal, Cecilia D. Treviño-Quintanilla, Ulises Sánchez-Santana, Enrique Cuan-Urquizo, Esmeralda Uribe-Lam

**Affiliations:** 1 Tecnologico de Monterrey, School of Engineering and Sciences, Queretaro 76130, Mexico; 2 Tecnologico de Monterrey, Institute of Advanced Materials for Sustainable Manufacturing, Queretaro 76130, Mexico; 3 Centro de Ingeniería y Desarrollo Industrial, Pie de la Cuesta 702, Desarrollo San Pablo, Queretaro 76130, Mexico

**Keywords:** mechanical characterization, continuous fiber reinforced composites, polymer matrix, additive manufacturing, Kevlar^®^

## Abstract

Additive manufacturing of composite materials is progressing in the world of 3D printing technologies; composite materials allow the combination of the physical and mechanical properties of two or more constituents to create a new material that meets the required properties of several applications. In this research, the impact of adding Kevlar^®^ reinforcement rings on the tensile and flexural properties of the Onyx™ (nylon with carbon fibers) matrix was analyzed. Parameters such as infill type, infill density and fiber volume percentage were controlled to determine the mechanical response in tensile and flexural tests of the additive manufactured composites. The tested composites showed an increment of four times the tensile modulus and 1.4 times the flexural modulus of pure Onyx™ matrix when compared with that of the Onyx™–Kevlar^®^. The experimental measurements demonstrated that Kevlar^®^ reinforcement rings can increase the tensile and flexural modulus of Onyx™–Kevlar^®^ composites using low fiber volume percentages (lower than 19% in both samples) and 50% of rectangular infill density. However, the appearance of some defects, such as delamination, was observed and should be further analyzed to obtain products that are errorless and can be reliable for real functions as in automotive or aeronautical industries.

## 1. Introduction

Composite materials have revolutionized the world of manufacturing processes with the ability of blending the mechanical and physical properties of the constituents, creating a new material [1]. Composite materials consist of two or more constituents, a soft and continuous matrix and a discontinuous and hard reinforcement. The matrix is responsible for distributing the loads applied along the reinforcements, and reinforcements withstand the loads [1,2]. The selection of constituents and the distribution gives the material specific mechanical properties such as tensile modulus, flexural modulus and impact resistance, among others [2].

Additive manufacturing (AM) encounters applications in the medical [3,4,5], automotive [6,7,8,9] or aerospace fields [10,11,12]. Due to industry 4.0, AM techniques are gradually becoming more important in the manufacturing industry because of the ability of their process to produce complex geometries and optimize the material volume used [13,14,15,16,17]. Different AM techniques have been used to produce composite materials; among this, fused filament fabrication (FFF) is the most frequently used [18]. The FFF process has been used to produce PLA–Kevlar^®^ composites [19,20], carbon–PEEK composites [21] or epoxy resin composites reinforced with carbon, glass and Kevlar^®^ reinforcement [22].

FFF processes include the continuous fiber reinforcement composites (CFRC) technique, which consists of extruding a thermoplastic matrix and reinforcing it with continuous fibers in certain layers. The matrices regularly used in this CFRC technique are polyamide-based thermoplastics, such as nylon and Onyx™, and the reinforcements frequently applied are carbon, glass and Kevlar^®^ (aramid) fibers [23]. The products obtained from CFRC technology exhibit mechanical properties similar to aluminum alloys [16,24] and inferior mechanical responses compared with traditionally manufactured composites [25].

Onyx™ is a thermoplastic matrix commonly used in CFRC processes to fabricate composite products; is a nylon-based material reinforced with chopped short carbon fibers; and offers high strength, toughness and chemical resistance for 3D-printed parts [26]. A typical reinforcement material used in the CFRC process is Kevlar^®^ fiber, which is the commercial name for aramid fiber, obtained from the synthesis of aromatic polyamides [2]. Kevlar^®^ fiber has a tensile modulus of 27 GPa and a flexural modulus of 26 GPa, and due to its high toughness, is used in ballistic, armor, energy absorption and high-velocity impact applications [19,26,27,28].

The printing parameters in the manufacturing process of CFRC products are crucial because they will have an impact in the resulting mechanical properties of these products [29]. The parameters that can be modified in a slicer software are the type of matrix, reinforcement fiber, fiber fill type, infill geometry, fiber infill orientation, fiber volume and fiber reinforcement rings; these parameters have been analyzed in order to study their effect on the mechanical properties and behavior of CFRC products [30,31].

The mechanical properties of 3D-printed composites are significantly influenced by fiber orientation and content [32]; for example, the out-of-plane shear strength of nylon–Kevlar^®^ composites increases when the size of the 3D-printed product increases due to the increment of fiber volume [33]. It was found in [34] that the interlaminar shear strength is lower in 3D-printed composites in comparison with traditionally manufactured composites because of the manufacturing process. The layer-by-layer process cannot create the required pressure to eliminate voids and spaces between fiber and matrix [35,36] like in traditional manufacturing techniques such as vacuum processes.

The response under tensile and flexural loading of nylon composites has been analyzed by different researchers [33,37,38,39,40,41,42,43,44,45] evaluating the effect of different fibers, such as carbon, glass and Kevlar^®^, on the properties of the composites. Heitkamp et al. [44] found an increment in the impact strength of nylon matrix when unidirectional aramid fiber volume was increased, but tensile and flexural strength was decreased when Kevlar^®^ fiber volume was increased. Mohammadizadeh and Fidan [39] added 60% of reinforcement and obtained an increment in tensile response of nylon matrix of 2.231% using carbon fiber, 1.703% using glass fiber and 1.404% using Kevlar^®^ fiber. The elastic modulus also showed an increment versus pure nylon matrix of 17.206% using carbon fiber, 7.206% using glass fiber and 6.001% using Kevlar^®^ fiber.

Giarmas et al. [41] studied nylon–glass and found that unidirectional glass fiber increases up to 141% and 432% the flexural strength and modulus, respectively. Saidane et al. [45] studied nylon composites, finding that the addition of unidirectional glass fiber gave an increment of 85% and 87% elastic modulus and tensile strength. Dickson et al. [46] evaluated the flexural response of nylon composites reinforced with carbon, glass and Kevlar^®^ fibers, obtaining higher flexural strength (250 MPa) with carbon than with glass (196 MPa) and Kevlar^®^ (125 MPa) fiber reinforcements. Korkees et al. studied nylon–carbon fiber results and showed an increment in flexural strength when the fiber volume was increased, from 30 MPa with 0% fiber volume to 450 MPa with 65% fiber volume [38].

Onyx™-based CFRC reinforced with carbon, glass and Kevlar^®^ fibers have been also analyzed to obtain their properties, such as flexural, tensile, compressive, shear and impact properties [26,29,47,48,49,50,51,52,53,54,55,56,57,58,59,60,61,62,63]. Bárnik et al. [51] found in pure Onyx™ samples that the higher ultimate force is obtained when the infill density is higher and triangular infill geometry is used. Ekoi et al. [53] established that carbon fibers deposited unidirectionally in Onyx™ matrix gave the maximum tensile strength (714 MPa), maximum flexural strength (407 MPa) and the maximum ultimate strength in fatigue tests with up to 200,000 cycles [53]. Parmiggiani et al. [50] evaluated the tensile and flexural properties of Onyx™–carbon composites, obtaining high tensile strength (566 MPa), stiffness (24.2 GPa) and flexural resistance (340 MPa) with unidirectional fiber orientation and 57% of fiber volume [50].

Cofaru et al. [60] analyzed the tensile properties of Onyx™ composites reinforced with carbon, glass and Kevlar^®^ fibers; the lowest strain was obtained using carbon fibers, followed by Kevlar^®^ and finally glass fibers, and the highest elastic modulus was obtained with carbon, followed by Kevlar^®^ and finally glass fibers. Papa et al. [26] analyzed Onyx™ reinforced with different carbon fiber directions, obtaining a higher response to forces using unidirectional fibers at 0°, in comparison with the 0°/90° arrangement. Ansari and Kamil [61] also tested the tensile properties of Onyx™ composites reinforced with carbon, glass and Kevlar^®^ fibers; the results showed that the fiber angle is the one that is responsible for the ultimate tensile strength behavior, and the highest tensile was obtained using unidirectional isotropic direction. Ojha et al. [63] observed that adding fibers would increase elastic modulus and stiffness but adding more than eight layers of fibers would result in a decrease in elastic modulus and stiffness.

The presented studies worked with different combinations and analyzed the mechanical response of a particular CFRC printing configuration because Markforged Inc. only reported the constituent materials properties separately. The capability of adding the desired proportions of matrix and fiber opens the possibility of varying printing parameters and obtaining the desired mechanical behavior of a CFRC product.

The use of an internal geometry in AM products results in lightweight products that can maintain certain mechanical properties, translating to material optimization. The impact of only using reinforcement rings in combination with rectangular infill geometry has not been studied profoundly. The presented studies use samples with solid or triangular infill and fiber reinforcement infill using different fiber orientations with their respective reinforcement rings. Therefore, this research focuses on the study of the impact of using rectangular infill geometry and only the Kevlar^®^ reinforcement rings, reducing the percentage of fiber (lower than 19% of fiber volume) and material utilized to create the Onyx™–Kevlar^®^ samples to obtain the mechanical properties and analyze the effect of adding the Kevlar^®^ reinforcement rings to Onyx™, for comparison with the obtained properties with pure Onyx™ results published by Markforged [64].

## 2. Materials and Methods

### 2.1. Materials

The CFRC samples were fabricated with Onyx™ as the thermoplastic matrix constituent and Kevlar^®^ as the fiber reinforcement constituent. Onyx™ and Kevlar^®^ fiber filaments have a diameter of 1.75 mm and 0.3 mm, respectively, and they were supplied by Markforged Inc., Watertown, MA, USA. The mechanical properties of Onyx™ and Kevlar^®^ filaments are described in Table 1.

### 2.2. Methods

#### 2.2.1. Printing of Onyx™–Kevlar^®^ Samples

**Figure 1:**
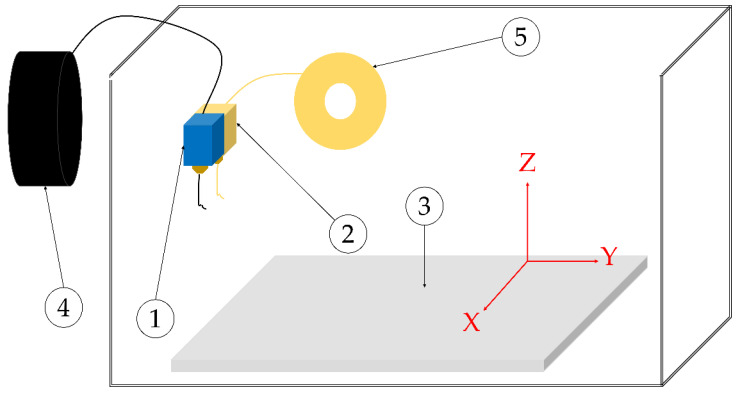
Scheme of Mark Two™ principal components. (1) Matrix nozzle. (2) Reinforcement nozzle. (3) Printing bed. (4) Matrix filament. (5) Reinforcement filament.

The samples were manufactured in a Mark Two™ 3D printer by Markforged Inc., Watertown, MA, USA. This printer works with a dual nozzle, as seen in Figure 1. The first nozzle deposits the thermoplastic matrix, and the second nozzle deposits the fiber reinforcement in the specified zones, substituting the infill geometry selected for the piece. The printer requires a special slicer software called “Eiger™”, by Markforged^®^, Inc. In this software, the printing parameters to achieve the 3D-printed samples are set.

**Figure 2:**
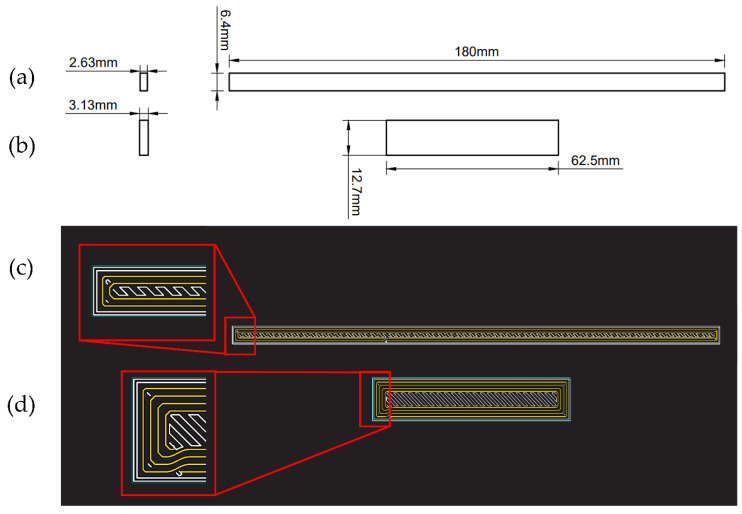
(**a**) Technical drawing of tension samples. (**b**) Technical drawing of bending samples. (**c**) Scheme of Kevlar^®^ reinforcement rings of tensile samples. (**d**) Scheme of Kevlar^®^ reinforcement rings of bending samples.

The 3D-printed samples were manufactured following the standards established, i.e., ASTM D3039 (tension) and ASTM D790 (bending tests). The dimensions of the samples are shown in Figure 2a (tension) and Figure 2b (bending). The Kevlar^®^ reinforcement rings are represented as yellow concentric rectangles in Figure 2c (tension) and Figure 2d (bending).

The testing method, the test standard, fiber orientation, infill geometry, fiber volume and reinforcement rings in the samples used in this research are summarized in Table 2.

**Figure 3:**
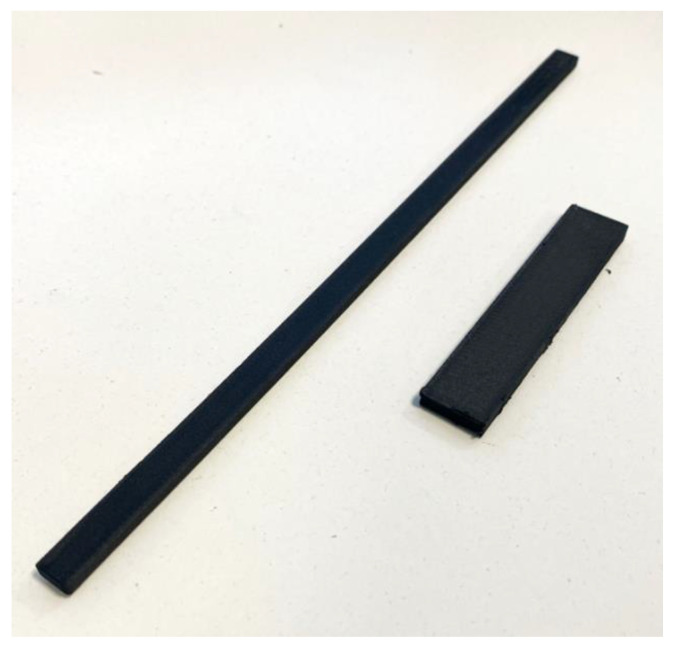
Samples for tensile (left) and bending (right) tests.

The samples used for tensile and bending tests are presented in Figure 3.

#### 2.2.2. Mechanical Analysis

**Figure 4:**
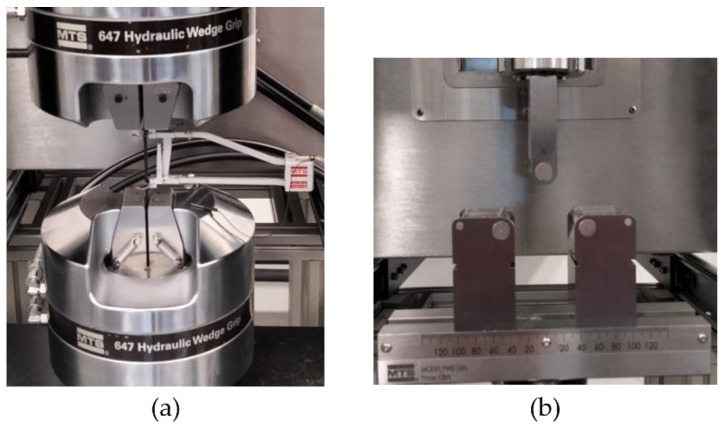
MTS Insight machine fixtures for (**a**) tensile and (**b**) bending tests at room temperature.

To characterize the mechanical properties of the Onyx™–Kevlar^®^ composite, tensile and flexural tests were performed in an MTS Insight machine by MTS Systems Corporation, Eden Prairie, MN, USA and an MTS-634.25 axial extensometer with 50 mm (2 in.) Gage Length was used to measure the strain in the specimens. Tensile tests were conducted following ASTM D3039 standards and flexural tests following ASTM D790 standards. Five repetitions of each mechanical test were performed to obtain reliable and repeatable results, as marked in ASTM D3039 and ASTM D790 standards. For tensile tests, the samples were placed between a pair of clamps, presented in Figure 4a. These clamps have a hydraulic system that allows the user to adjust the holding pressure to ensure the samples are clamping; the clamps were configured to apply a pressure of 150 psi to hold the tensile samples in place. To ensure a holding distance that would allow correct measurements in the tensile tests, prior trials were made to have the separation of the clamps that would secure the pieces in place and that would allow the rupture in a mid-zone of the CFRC samples; this distance was 80 mm between clamps. Then, a tensile load of 100 KN at a rate of 2 mm/min (as mentioned in ASTM D3039) was applied to the samples until failure at room temperature.

The tensile test allowed us to characterize the response of the composite to the load applied in the axial direction of the samples. The results of the tensile test were analyzed using the Hooke’s general law to obtain the elastic modulus of the composite material by using Equation (1):

(1) $$\sigma_{max}=E\varepsilon$$

where $\sigma_{max}$ is the maximum stress applied to the sample, $E_{T}$ is the elastic modulus of the composite material and ε is the elastic strain at $\sigma_{max}$, which was obtained using Equation (2),

(2) $$\varepsilon =\frac{\delta L}{L_{o}}=\frac{L_{f}-L_{o}}{L_{o}}$$

The $E_{T}$ of the Onyx™–Kevlar^®^ composite can be determined by solving for the slope in the linear section of the stress–strain curve using Equation (3),

(3) $$E_{T}=\frac{\sigma_{i+1}-\sigma_{i}}{\varepsilon_{i+1}-\varepsilon_{I}\;}$$

where i represents the position of the $i^{th}$ stress and strain values to be substituted in Equation (3).

The flexural test selected was a three-point bending test, as seen in Figure 4b. Before the tests were conducted, the samples were measured in order to obtain the values for the geometrical parameters used in Equation (4). The samples were placed in two supports that had a separation of 50 mm. The separation between supports was calculated by multiplying the samples’ width times 16, as mentioned in ASTM D695. Then, a load of 1 KN at a rate of 2 mm/min (as mentioned in ASTM D790) was applied at the center of the samples until failure at room temperature.

For bending test results, the flexural modulus $\left(E_{f}\right)$ can be obtained using Equation (4) [65].

(4) $$E_{f}=\frac{L^{3}\;m}{4bd^{3}}$$

where L is the span length, b is the sample width and d is the beam thickness. The values of *m* were obtained by finding the slope of the straight-line segment of load–displacement curves, using Equation (5) [65].

(5) $$m=\frac{Load_{i+1}-Load_{i}}{displacement_{i+1}-displacement_{i}\;}$$

where i represents the position of the $i^{th}$ load and displacement values to be substituted in Equation (5).

#### 2.2.3. Dehumidification Process

Mechanical characterization results can be affected by the humidity absorption of nylon polymer and interlayer zone imperfections [48]. Onyx™ is made of nylon; it absorbs humidity from ambient [66,67], which is why it is stored in a dry-box in the whole manufacturing process [50,53,59,63]. The printed samples require a dehumidification that is achieved with a dry thermal process in an electrical furnace to eliminate the humidity absorbed during the printing process [42,48,50]. The electrical furnace used was TE-M20AT from Terlab, Escondido, CA, USA. The samples were heated at 70 °C for 4 h to eliminate the humidity in Onyx™. Some researchers have stored the samples in sealed bags to prevent changes caused by the environment [60]; in this research, the treated samples were stored in a desiccator to reduce the mechanical test variation results produced by moisture in the samples [67].

#### 2.2.4. Density Analysis

Onyx™–Kevlar^®^ composite samples were subjected to a density analysis in a Metter Toledo XS104 Analytical Balance at room temperature. To determine the real density of the samples, standard ASTM D792 was followed using the water displacement method. The density of the tested samples was calculated to determine the specific tensile and flexural modulus considering the fiber volume percentage and infill density of the samples.

## 3. Results and Discussion

### 3.1. Tensile Properties

**Figure 5:**
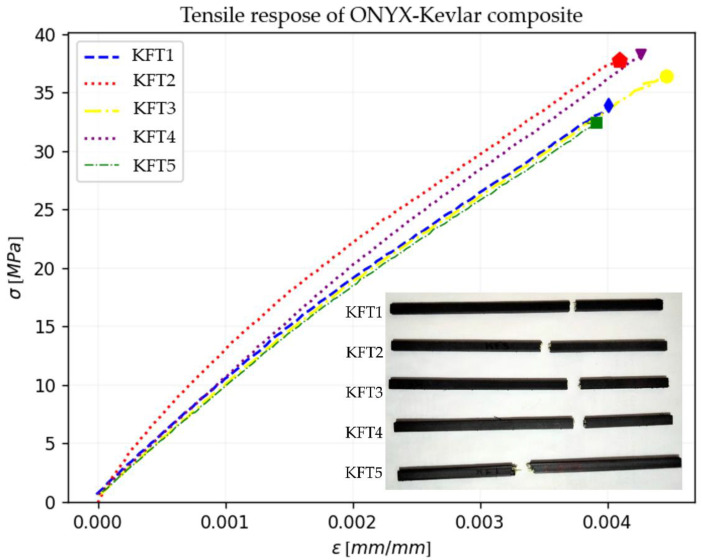
Tensile test stress–strain curves and samples after failure (KFT1, KFT2, KFT3, KFT4 and KFT5 images showed are the actual samples for tensile tests).

The tensile properties of the five 3D-printed composite samples are represented in Figure 5 where the strain–stress curves are plotted. Figure 5 also displays a picture of the samples used and the failure mechanism at $\sigma_{max}$. The samples’ designation is KFT, where KFT means Kevlar^®^ Fiber Tensile, followed by the sample test number from one to five.

The results of the tensile test, summarized in Table 3, are the individual elastic properties of Onyx™–Kevlar^®^ composites tested in this research and the mean values.

The Kevlar^®^ reinforcement rings showed an increment in tensile response. All the samples presented higher values, with a mean value of 9.57 GPa in tensile modulus, in comparison with the 2.4 GPa tensile modulus of pure Onyx™ published by Markforged [64]. This increment is the result of the arrangement of the fibers unidirectionally aligned parallel to the same axis to which the tensile force was applied, as mentioned in [45,50,53,61]. The unidirectional fibers withstand higher tensile stress in comparison with different orientation of reinforcement fibers. This is due to the distribution of stresses along the axis on which the fibers are deposited, having minimal or no significant shear stresses to affect tensile performance.

The 3D printing process has natural variations in the printed products. For example, the KFT5 sample showed a lower maximum strain compared with the other tensile samples; this can be attributed to defects in the manufacture of the part, since it is known that the technologies for creating CFRC are not yet reliable enough to have a product that always fulfills the same physical and mechanical characteristics due to printing defects that can lead to premature failure [29,56,68].

The obtained $E_{T}$ was compared with other research found in the literature; the different CFRC samples tested and the obtained $E_{T}$ are presented in Table 4. The obtained $E_{T}$ was achieved with a reduced fiber volume in comparison with other studies [39,44,50,53,60,61,63], who obtained their maximum results using 25.8% to 60% of fiber volume in comparison with the 18.77% used in this research.

### 3.2. Flexural Properties

**Figure 6:**
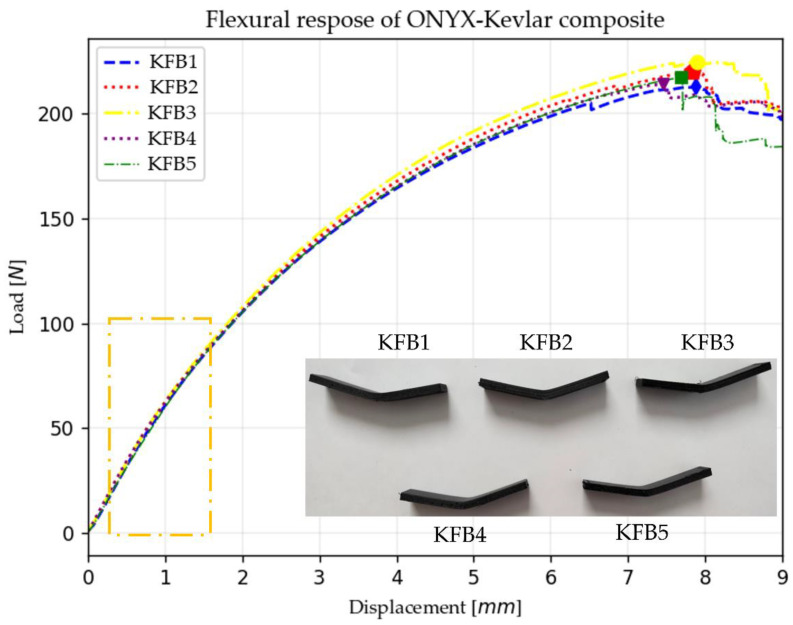
Bending test load–displacement curves, linear zone considered for flexural modulus, marked as a rectangle, and samples after failure (KFB1, KFB2, KFB3, KFB4 and KFB5 images shown are the actual samples for bending tests).

The flexural test results are shown in Figure 6 plotted in a load–displacement curve, with a picture of the samples used and the deformation after maximum displacement was reached. The samples’ designation is KFB, where KFB means Kevlar^®^ Fiber Bending, followed by the sample test number from one to five.

The results of the flexural test, summarized in Table 5, are the individual flexural properties of Onyx™–Kevlar^®^ composites tested in this research and their mean values.

The Kevlar^®^ reinforcement rings also have a positive increasing effect on the flexural modulus of the Onyx™–Kevlar^®^ composite. The flexural modulus increases from 3.0 GPa for pure Onyx™ [64] to 4.11 GPa in mean for Onyx™ with Kevlar^®^ reinforcement rings. This increment is also due to the direction of the fibers, since the fibers along the length of the samples help to distribute the stresses along the sample geometry, having a distribution from the center of the sample, where the load is applied, towards the supports of the three point bending test.

The obtained $E_{F}$, indicated with a rectangle in Figure 6, was compared with other research found in the literature. The different CFRC samples tested and the obtained $E_{F}$ are presented in Table 6. The bending properties of Onyx™–Kevlar^®^ are not as high as Onyx™–carbon products, where the $E_{T}$ is higher than 24 GPa, but it was demonstrated that the addition of Kevlar^®^ reinforcement rings can improve the mechanical response of CFRC products to bending forces.

### 3.3. Printing Defects

**Figure 7:**
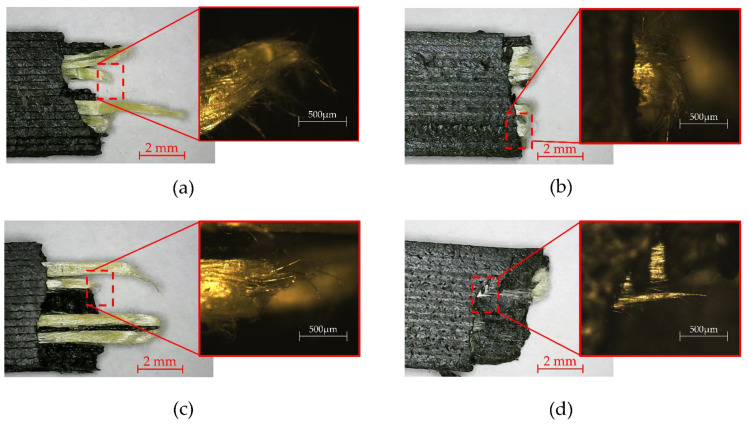
ASTM D3039 tensile failure mechanisms and composite behavior presented in Onyx™–Kevlar^®^ samples and microscopic view. (**a**) AGM with fiber debonding, (**b**) LGM with fiber-matrix breakage, (**c**) DGM with matrix breakage and (**d**) DGM with fiber pull-out.

After experimental tests, the samples were inspected; different type of failures and repetitive printing defects in tensile and flexural samples were found. In Figure 7, the different types of failures presented in tensile test samples can be seen. The failure modes were compared with the ASTM D3039 failure modes atlas to determine a qualitative standardized comparison. The samples presented in the matrix present a premature failure due to delamination, as in [52], leaving all the load to the Kevlar^®^ fibers until they also reach and overpass the $\sigma_{max}$ of fibers.

**Figure 8:**
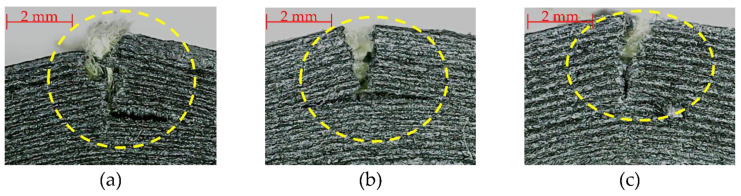
Bending failures in Onyx™–Kevlar^®^ composites: (**a**) matrix breakage, (**b**) delamination and (**c**) crack propagation. The areas with failures are marked within the yellow dotted circle.

In Figure 8, the different types of defects and failure mechanisms in flexural samples are shown. In these samples, fracture occurs in the same direction that the load was applied. In the samples KFB2, KFB3 and KFB4, delamination was present after maximum deflection was reached. In the samples KFB2 and KFB4, the fibers came out of the matrix and were exposed to the environment due to poor bonding between the matrix and fibers.

The Kevlar^®^ fibers in the tensile test suffer pull-out from the matrix, as seen in Figure 7a,b, due to an interlaminar failure, as mentioned in [46]. The failures and defects presented in Figure 7 and Figure 8 show that the matrix and fibers have poor bonding; this can be the result of air bubbles or voids in the samples, as mentioned in [41,52,53,70]. The delamination presented in Figure 8 is described in the literature as poor adhesion of the different layers of matrix and reinforcement, an effect that is typically present in the FFF and CFRC processes [41]. The defects and failure mechanisms that were observed in this research are similar to the ones reported in [38,39,56].

CFRC samples can be affected by the presence of voids, lack of molding conditions and low impregnation causing a low adhesion between the matrix and fibers, resulting in lower mechanical properties compared with traditionally manufactured composites [34,71,72]. Voids can be reduced using the postprocessing technique presented in [68], which consists of placing the 3D-printed composite into a mold and applying heat and pressure to it, to reduce the voids between matrix filaments and matrix–fiber bonding. The poor matrix interface will cause a reduction in mechanical performance [70].

Another effect that can impact the mechanical response in the samples used in this research is the fiber waviness, a phenomenon that creates variations in the out-of-plane zones where the continuous fiber is deposited, altering the distribution of loads along the fibers in the different layers of the 3D-printed product [73,74].

### 3.4. Density Test

Three representative samples of the five used in tensile and flexural tests were subjected to density analysis to determine the specific elastic and flexural modulus of the samples considering matrix, reinforcement and infill selected for the Onyx™–Kevlar^®^ composite. The results of these density analyses were used to calculate the specific elastic modulus and the specific flexural modulus of the Onyx™–Kevlar^®^ composites. The mean density value for tensile samples was $1.028\;g/{\mathrm{cm}}^{3}$ and the mean density value for bending samples was $1.033\;g/{\mathrm{cm}}^{3}$. These results gave a specific elastic modulus of $9.31\;\mathrm{GPa}\cdot {\mathrm{cm}}^{3}/g$ and a specific flexural modulus of $3.98\;\mathrm{GPa}\cdot {\mathrm{cm}}^{3}/g$. The variation in density can be attributed to variation in the printing process as over or under extrusion of the matrix materials, even though the samples were printed using the same equipment.

## 4. Conclusions

Tensile and bending tests were carried out to determine the effect of fiber reinforcement rings on the tensile and flexural properties of Onyx™–Kevlar^®^ 3D-printed composites.

- The addition of 18.77% of fiber volume as Kevlar^®^ reinforcement rings to Onyx™ with 50% of rectangular infill resulted in an increment of almost 400% of pure Onyx™ elastic modulus. A value of 9.57 GPa was obtained with lower fiber volume compared with other studies that go from 1.3 GPa to 4.95 GPa using 25.8% to 60% of fiber volume.
- The addition of 17.25% of fiber volume as Kevlar^®^ reinforcement rings to Onyx™ with 50% of rectangular infill resulted in an increment of almost 140% of pure Onyx™ flexural modulus. A flexural modulus of (4.11 GPa) was obtained, and similar results (4.73 GPa) were obtained using 19% of fiber volume and triangular infill.
- A specific elastic modulus of $9.31\;\mathrm{GPa}\cdot {\mathrm{cm}}^{3}/g$ was obtained and a specific flexural modulus of $3.98\;\mathrm{GPa}\cdot {\mathrm{cm}}^{3}/g$ was obtained for Onyx™–Kevlar^®^ CFRC.

After experimental tests, some defects were registered; the effect of defects on composite printed parts requires further investigation to improve surface quality of the printing samples. In the sample fracture mechanism, a weak adhesion in the interlayer zone of the matrix and reinforcement was observed, so further investigations are needed to control the interlayer adhesion. The presence of the described defects in the CFRC samples leads to the conclusion that further analysis needs to be conducted in the FFF technology for composite materials, since the defects decrease the reliability of CFRC products and thus diminish its early use in the industry as a replacement for traditionally manufactured composite materials.

With the presented results, it can be concluded that the reinforcement rings occupied a low volume in the 3D-printed product geometry. These reinforcement rings can be used in industrial applications such as aeronautics or the automotive field, where lightweight structures are designed to withstand the mechanical stresses to which products in these fields are subjected. However, more research is required in the study of reinforcement rings, considering more levels in the analysis, by analyzing the continuous addition of reinforcement rings used with the aim of optimizing the materials used but fulfilling the required mechanical properties for certain applications.

The printing parameters that the Mark Two printer have are limited for the user to modify them; thus, more options to vary in printing parameters can help in the improvement of 3D-printed composite materials and achieve similar mechanical properties of traditionally made composites.

## Figures and Tables

**Table 1:** Mechanical properties of Onyx™ filament [64].

Property	Onyx™	Kevlar^®^
Tensile Modulus [GPa]	2.4	21
Tensile Strain at Break [%]	25	2.7
Flexural Strength [MPa]	71	240
Flexural Modulus [GPa]	3	26
Density [g/cm^3^]	1.2	1.2

**Table 2:** Sample configuration for mechanical characterization of Onyx™–Kevlar^®^ composite.

Testing Method	ASTM Standard	Fiber Orientation	Infill Geometry	Fiber Volume (vol%)	Fiber Reinforcement Rings
Tension	D3039	Concentric	Rectangular	18.77%	2
Bending	D790	Concentric	Rectangular	17.25%	4

**Table 3:** Tensile test results of Onyx™–Kevlar^®^ 3D-printed composites.

Sample	Max Stress (MPa)	$\mathrm{Max}\;\mathrm{Strain}\;\left(\frac{mm}{mm}\right)$	$E_{T}$ (GPa)
KFT1	33.93	0.004	9.17
KFT2	37.75	0.0041	10.47
KFT3	36.43	0.0045	9.22
KFT4	38.25	0.0043	9.84
KFT5	32.42	0.0039	9.14
Mean	35.756	0.00416	9.57

**Table 4:** Tensile modulus comparison with other CFRC research.

Research	CFRC	$E_{T}$ [GPa]
This research	Onyx™–Kevlar^®^	9.57
Heitkamp et al. [44]	Nylon–Kevlar^®^	6.42
Mohammadizadeh et al. [39]	Nylon–Kevlar^®^	18.12
Cofaru et al. [60]	Onyx™–Kevlar^®^	4.95
Ojha et al. [63]	Onyx™–Kevlar^®^	1.3
Ansari [61]	Onyx™–Kevlar^®^	1.53

**Table 5:** Bending test results of Onyx™–Kevlar^®^ 3D-printed composites.

Sample	Max Load (N)	Max Displacement (mm)	$\mathrm{Slope}\;\left(\frac{N}{mm}\right)$	$E_{B}$ (MPa)
KFB1	212.64	7.88	52.51	4.12
KFB2	219.93	7.84	52.31	4.10
KFB3	224.62	7.90	52.72	4.13
KFB4	213.85	7.46	50.99	3.99
KFB5	217.30	7.70	53.71	4.19
Mean	217.67	7.75	52.45	4.11

**Table 6:** Flexural modulus comparison with other CFRC research.

Research	CFRC	$E_{F}$ [GPa]
This research	Onyx™–Kevlar^®^	4.11
Heitkamp et al. [44]	Nylon–Kevlar^®^	7.92
Parmiggiani et al. [50]	Onyx™–Carbon	24.39
Chacón et al. [69]	Nylon–Kevlar^®^	0.5
Pertuz-Comas et al. [62]	Onyx™–Kevlar^®^	4.73

## Data Availability

The data presented in this study are available on request from the corresponding author.
